# Dietary Modulation of Metabolic Health: From Bioactive Compounds to Personalized Nutrition

**DOI:** 10.3390/metabo15090624

**Published:** 2025-09-19

**Authors:** Aleksandra Leziak, Julia Lipina, Magdalena Reclik, Piotr Kocelak

**Affiliations:** 1 Students’ Scientific Association, Department of Pathophysiology, Medical Univeristy of Sielsia, 40-752 Katowice, Poland; s82952@365.sum.edu.pl (A.L.);; 2 Department of Pathophysiology, Medical University of Silesia, 40-752 Katowice, Poland

**Keywords:** bioactive compounds, functional food, metabolic health, obesity, metabolomic, personalized nutrition

## Abstract

Metabolic health is a dynamic equilibrium influenced by diet and lifestyle. This review synthesizes evidence on how specific dietary patterns and bioactive nutrients modulate metabolic disorders. Diets like the Mediterranean and DASH plans consistently improve cardiometabolic markers: a Mediterranean diet can halve metabolic syndrome prevalence (~52% reduction) in as little as 6 months, while the DASH diet typically lowers systolic blood pressure by ~5–7 mmHg and modestly improves lipid profiles (LDL-C by ~3–5 mg/dL). Plant-based diets (vegetarian/vegan) are associated with lower BMI, improved insulin sensitivity, and reduced inflammation. Ketogenic diets induce rapid weight loss (~12% body weight vs. 4% on control diets) and improve glycemic control (reducing HbA1c and triglycerides), though long-term effects (elevated LDL) warrant caution. Bioactive compounds present in these diets play critical roles: polyphenols improve insulin signaling and reduce oxidative stress (resveratrol supplementation reduced HOMA-IR by ~0.5 units and fasting glucose by ~0.3 mmol/L); omega-3 fatty acids (fish oil) reduce triglycerides by ~25–30% and inflammation; and probiotic interventions modestly enhance glycemic control (lowering HOMA-IR and HbA1c) and gut health. Personalized nutrition approaches, which account for genetic and microbiome differences, are emerging to maximize these benefits. In conclusion, evidence-based dietary strategies rich in fiber, unsaturated fats, and phytochemicals can substantially improve metabolic health outcomes, underscoring the potential of tailored nutrition in preventing and managing obesity-related metabolic disorders.

## 1. Introduction

Metabolic health refers to a physiological state in which key metabolic systems function efficiently and in coordination, effectively managing energy production, nutrient absorption, utilization, and internal homeostasis [1]. Such optimal metabolic function typically involves stable physiological parameters, such as blood glucose levels, lipid profiles, blood pressure, and an appropriate waist-to-hip ratio (WHR), achieved without pharmacological intervention [2]. The capacity for metabolic flexibility, allowing adaptation to fluctuating energy demands and environmental stimuli, significantly reduces the risk of chronic metabolic diseases [3].

Metabolic disorders have increasingly become prevalent globally, driven predominantly by modern lifestyle factors such as sedentary behavior, unhealthy dietary habits, and chronic psychosocial stress. Collectively, these factors contribute to widespread metabolic dysregulation, highlighting an urgent need for prioritizing metabolic health in global public health strategies [4].

Excessive adiposity, characterized by abnormal or disproportionate body fat accumulation, commonly manifests as overweight or obesity [5]. While traditionally associated with adverse metabolic outcomes, recent research recognizes a distinct phenotype, termed metabolically healthy obesity (MHO), wherein individuals maintain a high body mass index (BMI) but do not exhibit typical metabolic syndrome (MetS) markers [6,7]. Observational studies indicate that a subgroup within the obese population might display resilience or reduced susceptibility to cardiometabolic diseases, challenging the universally assumed correlation between BMI and metabolic risk [8]. Conversely, metabolically unhealthy phenotypes can occur in individuals with normal BMI who display insulin resistance (IR), dyslipidemia, or chronic inflammation, underscoring the complexity of metabolic health beyond body weight metrics alone [9].

The global increase in metabolic dysfunctions, particularly those related to obesity, has been significantly influenced by shifts in dietary behaviors, including consumption of processed foods high in sugars and unhealthy fats, coupled with declining physical activity levels inherent to contemporary urban lifestyles [10,11]. In 2022, 2.5 billion adults were overweight. Of these, 890 million were living with obesity [12]. MetS, characterized by a clustering of elevated blood pressure, dyslipidemia, IR, and abdominal obesity, poses substantial health and social challenges, notably in countries such as Poland [13]. Current estimates indicate that MetS affects approximately 20–25% of the global population, with higher prevalence rates observed in certain populations or regions, significantly burdening healthcare systems due to the heightened risk of cardiovascular disease, type 2 diabetes (T2D), and other chronic illnesses [14,15].

Diet emerges as a crucial, modifiable determinant of metabolic health [16]. Beyond caloric intake alone, dietary quality—encompassing macronutrient composition, fiber content, and micronutrient density—plays an integral role in metabolic regulation [17]. Dietary interventions targeting whole, minimally processed, plant-based foods, lean proteins, and healthy fats demonstrate potential for preventing, delaying, or reversing metabolic abnormalities such as impaired insulin function, dyslipidemia, and chronic inflammation [18,19].

Given the escalating burden of obesity-related, non-communicable diseases such as T2D [20], cardiovascular disease [21], and metabolic-associated fatty liver disease (MAFLD) [22], identifying dietary strategies to enhance metabolic outcomes is paramount. In 2025, approximately 589 million individuals were affected by T2D [23]. Recent advances have highlighted the influence of dietary patterns, nutrient composition, and bioactive dietary compounds on modulating body composition and essential metabolic pathways, including insulin signaling, lipid homeostasis, inflammation, oxidative stress, and gut microbiota [24,25].

Furthermore, developments in biomarker identification and metabolomics are offering innovative avenues for tailored dietary interventions and personalized nutritional strategies [26]. This review systematically synthesizes current evidence concerning diet, metabolic health, and adiposity. We begin by outlining key biomarkers and physiological pathways critical for metabolic assessment, followed by an evaluation of specific dietary patterns and bioactive components and their roles in metabolic regulation. Additionally, we explore the implications of advances in metabolomics, dietary supplements, and functional foods, and address challenges such as inter-individual variability in dietary response. Ultimately, this review highlights avenues for future research and provides practical insights relevant to clinical practice.

We will first examine evidence from dietary patterns known to improve metabolic health, followed by a discussion of bioactive food components and their molecular mechanisms. Finally, we address emerging approaches in personalized nutrition and biomarker-driven strategies.

## 2. Methodology

To prepare this narrative review, a non-systematic literature search was conducted focusing on current scientific evidence related to diet, metabolic health, and the role of bioactive food compounds. The search was performed using two major databases: PubMed and Embase, including publications available up to June 2025. Articles of various types were considered, including review papers, clinical trials, observational studies, and experimental research. The search strategy involved a combination of English keywords, such as “nutrition,” “eating habits,” “diet,” “bioactive compounds,” “functional foods,” and “metabolic health,”. The selection of sources was based on their relevance, clinical significance, and thematic alignment with the scope of this review. Special emphasis was placed on literature addressing dietary mechanisms and their effects on metabolic biomarkers, which formed the conceptual foundation for subsequent analysis.

## 3. Metabolic Health: Key Biomarkers and Pathways

The assessment of metabolic health requires a comprehensive evaluation of specific biomarkers indicative of the functional integrity of metabolic processes [26,27]. These biomarkers provide insight into the current metabolic state and serve as predictive tools for the risk of developing chronic non-communicable diseases such as T2D [26], cardiovascular diseases , and MAFLD [28,29,30]. Moreover, these biomarkers are vital for evaluating the efficacy of dietary and lifestyle interventions aimed at improving metabolic health [31]. The primary categories of metabolic biomarkers encompass indicators of body composition, insulin sensitivity, lipid metabolism, and systemic inflammation [32]. Key biomarkers reflecting these functions include fasting glucose, insulin, HbA1c, lipid profile, CRP, adipokines, liver enzymes, and microbiota-derived metabolites like SCFAs. These biomarkers are significantly influenced by bioactive compounds—such as polyphenols, flavonoids, carotenoids, and fiber—found in plant-based foods. These compounds modulate inflammation, oxidative stress, lipid levels, and insulin sensitivity, often via effects on gut microbiota. Dietary patterns rich in these substances, like the Mediterranean diet, consistently improve metabolic biomarkers, emphasizing the value of mechanistic insights for targeted nutrition strategies.

### 3.1. Body Composition

Although BMI remains widely used as a general obesity indicator, it does not distinguish between lean and fat mass, nor does it offer insights into fat distribution [33]. More precise measures, including waist circumference (WC), WHR, and advanced imaging techniques such as dual-energy X-ray absorptiometry (DEXA) or magnetic resonance imaging (MRI), facilitate a more accurate assessment of central or visceral adiposity [34]. Visceral fat, deposited around internal organs, is metabolically active and closely linked to IR and chronic inflammation, whereas subcutaneous fat has a comparatively less detrimental metabolic impact [35].

Body composition is crucial to overall health, physical functioning, and longevity, influenced by genetics, environmental factors, dietary habits, and physical activity. Accurate assessment of body composition allows healthcare professionals to evaluate nutritional status and monitor physiological responses to dietary or therapeutic interventions [36]. Aging typically involves an increase in adiposity and a concurrent decrease in lean mass, notably skeletal muscle and bone density [37]. Elevated body fat percentages correlate strongly with increased risk for cardiometabolic diseases such as cardiovascular disease, T2D, certain cancers, and heightened all-cause mortality [38]. Additionally, loss of skeletal muscle (sarcopenia) and reduced bone mineral density (osteopenia and osteoporosis) significantly contribute to frailty and diminished functional capacity [39].

Undernutrition exacerbates medical conditions and remains an underrecognized clinical concern. Relying solely on weight or BMI fails to distinguish between fat and fat-free mass, potentially masking their distinct contributions to disease risk [40]. Therefore, healthcare professionals must have a comprehensive understanding of advanced body composition assessment tools, including their strengths and limitations, to inform evidence-based clinical decisions.

Metabolically healthy obesity (MHO) refers to individuals with a high BMI but without typical metabolic abnormalities such as dyslipidemia, hypertension, or insulin resistance. This phenomenon presents a challenge to the traditional use of BMI as a proxy for metabolic health, revealing its limitations in capturing the functional and molecular status of an individual’s metabolism.

From a public health perspective, the recognition of MHO necessitates a shift away from weight-centric models toward metabolic profiling, which considers factors such as inflammation, insulin sensitivity, and hepatic fat content. While MHO individuals may have lower immediate cardiometabolic risk, longitudinal studies show they are still at higher risk than metabolically healthy normal-weight individuals, especially if their health status changes over time.

This contradicts conventional wisdom and urges re-evaluation of screening and intervention strategies, emphasizing comprehensive biomarker assessment over BMI alone. Policies should therefore promote early detection of metabolic dysfunctions in both obese and normal-weight populations through non-invasive biomarker panels and personalized lifestyle interventions.

#### 3.1.1. Insulin Sensitivity and Insulin Resistance

IR, characterized by diminished cellular responsiveness to insulin, is central to metabolic dysfunction and contributes directly to hyperglycemia and T2D pathogenesis [41]. Indicators frequently employed to evaluate insulin sensitivity include fasting blood glucose (FBG), fasting blood insulin (FBI), homeostasis model assessment of insulin resistance (HOMA-IR), and oral glucose tolerance testing [42].

Clinically, IR is identified when elevated insulin levels are necessary to maintain normoglycemia, serving as a robust predictor of T2D. IR commonly coexists with impaired glucose tolerance, visceral adiposity, and other MetS components [43,44]. Furthermore, IR is associated with various metabolic disturbances and elevated risk for conditions such as polycystic ovary syndrome (PCOS), MAFLD, and cardiovascular disease [45,46]. These conditions often manifest as compensatory hyperinsulinemia resulting from reduced insulin sensitivity.

Branched-chain amino acids (BCAAs), particularly leucine, isoleucine, and valine, influence insulin sensitivity significantly. Elevated circulating BCAAs are documented in both insulin-resistant individuals and rodent models. Increased dietary intake of these amino acids correlates with an 11–13% heightened risk of T2D [47]. Conversely, reducing dietary BCAAs has demonstrated improvements in glucose tolerance, diminished β-cell metabolic stress, and optimized body composition [48].

#### 3.1.2. Lipid Profile

Circulating lipids represent another pivotal aspect of metabolic health assessment. A standard lipid profile measures total cholesterol (TCh), high-density lipoprotein cholesterol (HDL-C), low-density lipoprotein cholesterol (LDL-C), and triglycerides (TGs) [49]. Obesity-associated atherogenic dyslipidemia is typified by elevated TGs, reduced HDL-C, and increased small, dense LDL (sdLDL) particles [50]. This lipid pattern substantially raises cardiovascular risk [51].

While some dyslipidemia have monogenic origins, most are polygenic and exacerbated by secondary factors such as obesity and T2D [52]. Dyslipidemia associated with obesity typically includes elevated levels of TGs, very-low-density lipoproteins (VLDL), apolipoprotein B (apoB), and non-HDL-C, combined with decreased HDL-C and apolipoprotein A-I (apoA-I). Although LDL-C may appear normal or slightly elevated, a shift toward sdLDL dominance increases total LDL-C particle number, heightening atherogenic risk [53,54].

#### 3.1.3. Inflammatory Markers

Obesity is frequently associated with chronic, low-grade inflammation, significantly contributing to IR. Visceral adipose tissue functions as an endocrine organ, secreting pro-inflammatory cytokines that disrupt insulin signaling and promote systemic metabolic disturbances [55]. Increased infiltration of pro-inflammatory macrophages, particularly M1-like subtypes, is characteristic of obesity-associated inflammation [56,57]. Compared to MHO, metabolically unhealthy obesity (MUO) features greater macrophage infiltration and a pro-inflammatory T-cell profile characterized by increased Th17 and Th22 cells alongside reduced anti-inflammatory Th2 cells [58].

Prominent inflammatory biomarkers include high-sensitivity C-reactive protein (hs-CRP), interleukin (IL)-6, and tumor necrosis factor-alpha (TNF-α) [59,60,61]. Elevated CRP, IL-6, TNF-α, and plasminogen activator inhibitor-1 are commonly observed in MUO versus MHO, correlating with increased MetS risk and associated complications [62,63]. Although subtle variations in cytokine expression and biomarker levels challenge the exclusivity of adipose-derived cytokines in driving IR, emerging evidence suggests that macrophage-derived exosomes may also mediate immune-associated metabolic dysregulation [64].

### 3.2. Dietary Responsiveness of Metabolic Biomarkers

The practical utility of metabolic biomarkers largely stems from their sensitivity to dietary interventions. Regular monitoring of insulin sensitivity, lipid profiles, inflammatory markers, and body composition facilitates both the evaluation and personalization of nutritional strategies tailored to individual metabolic profiles [65]. Certain biomarkers, including TGs and FBI, respond particularly rapidly to dietary changes, rendering them valuable for short-term monitoring and assessment of dietary compliance [26,66].

At a mechanistic level, dietary nutrients modulate critical metabolic pathways through diverse biological processes. These include regulating insulin signaling, influencing gene expression related to lipid metabolism, reducing oxidative stress, and altering gut microbiota composition [67,68]. However, substantial interindividual variability exists in response to dietary interventions, driven by genetic, epigenetic, and microbiome differences, highlighting the necessity for personalized nutrition approaches.

Ultimately, improvements in these responsive metabolic biomarkers correlate directly with reduced risk for cardiometabolic diseases, underscoring their relevance in both preventative and therapeutic clinical practice [69,70].

### 3.3. Metabolomics in Personalized Nutrition: Opportunities and Challenges

Metabolomics offers promising opportunities in personalized nutrition by enabling precise dietary recommendations based on individual metabolic profiles, allowing early detection of metabolic dysfunctions, and identifying responders to specific bioactive compounds. It also supports real-time monitoring of dietary interventions. However, challenges such as high costs, complex data interpretation, limited clinical accessibility, and ethical concerns around data privacy hinder its widespread application. Despite these limitations, the integration of metabolomics into nutrition strategies holds strong potential for improving metabolic health outcomes, as supported by current research on bioactive compounds’ mechanisms of action.

## 4. The Influence of Dietary Patterns on Metabolic Health

In response to the growing prevalence of excess body weight and related metabolic consequences, multiple dietary strategies have been intensively studied in recent years. Given the limitations and risks associated with pharmacological interventions, there is increased interest in non-pharmacological, diet-based approaches for enhancing metabolic health. The most extensively evaluated dietary patterns include the Mediterranean Diet (MedDiet), DASH (Dietary Approaches to Stop Hypertension) diet, ketogenic and low-carbohydrate diets, and plant-based dietary approaches, all demonstrating various metabolic benefits. Minimally processed food, the basis of the above dietary patterns, stands in opposition to typical components of Western diets and supports metabolic homeostasis. In contrast, Western diets promote chronic inflammation, oxidative stress, dyslipidemia and IR.

### 4.1. Western Diet

The Western diet is characterized by high caloric intake and frequent consumption of energy-dense, ultra-processed foods rich in sugar, salt, and fat, strongly associated with negative metabolic outcomes [24]. This dietary pattern promotes gut inflammation via adverse microbiome interactions and contributes to endotoxemia [24,71]. Low phytochemical and fiber intake exacerbates systemic inflammation, which may lead to anxiety, disordered eating, hyperphagia, and disrupted energy regulation—all of which contribute to obesity [24]. Western diets have been linked to increased BMI, body fat, IR, TG levels, and hepatic steatosis [72].

A study conducted by Schönknecht YB et al. [73] revealed a highly negative impact of Western dietary meals on antioxidant status. High-carbohydrate Western diets contribute to sustained elevations in insulin and glucose levels, while high-fat variants lead to elevated TG concentrations, which together disturb metabolic health and lead to the onset and progression of metabolic conditions, such as cardiovascular disease, T2D and MAFLD.

### 4.2. Mediterranean Diet

Dietary patterns significantly influence metabolic biomarkers. The MedDiet, characterized by high consumption of vegetables, legumes, fruits, whole grains, olive oil, nuts, and fish, has been extensively researched due to its significant positive health impacts, including glycemic control, improvement in lipid metabolism, and inflammatory markers [74,75]. Additionally, this diet involves moderate wine consumption and limited intake of red meat, dairy products, and sweets, further contributing to its metabolic benefits [75]. This dietary pattern is rich in fiber, antioxidants, and bioactive compounds, and typically involves low-energy-density foods, promoting satiety and facilitating caloric reduction [76,77]. Traditional Mediterranean eating habits involve shared family meals, which further contribute to overall dietary quality and lower caloric intake. Clinical evidence, notably from the Lyon Diet Heart Study, demonstrates that a MedDiet, rich in alpha-linolenic acid, significantly reduces cardiovascular events and mortality in secondary prevention contexts [78]. Furthermore, studies highlight the diet’s effectiveness in improving systolic and diastolic blood pressure (SBP and DBP), WHR, body weight, and body composition among adults diagnosed with MetS [79]. Marked improvements in glucose metabolism, lipid profiles, inflammation, and MetS regression have also been documented [80], and in that Italian trial, it was reported a ~52% reduction in metabolic syndrome prevalence after 6 months on a Mediterranean diet [80].

Furthermore, in clinical studies, high adherence to the MedDiet improved multiple metabolic syndrome components, and MedDiet interventions have demonstrated significant drops in blood pressure (≈5 mmHg) and fasting insulin, leading to improved HOMA-IR and regression of metabolic syndrome in adults. A 6-week transition from a Western diet to a MedDiet improves postprandial glucose levels and significantly lowers FBI and HOMA-IR [81]. Avoiding Western dietary patterns has been associated with improved lipid profiles and lower incidence of cardiovascular events [82], with the best outcomes observed when saturated animal fats are replaced by polyunsaturated fats—an effect comparable to that of statin therapy [83]. Implementing MedDiet interventions among overweight adolescents has resulted in substantial improvements in weight management, WC, SBP, FBG, insulin sensitivity, lipid profiles, and reduction in inflammatory markers like hs-CRP and IL-6 [84,85]. Adopting a MedDiet may be particularly relevant. Poor dietary habits and environmental influences have contributed to the increased prevalence of MetS in pediatric populations [86]. Additionally, adherence to a MedDiet in youth with obesity and MAFLD enhanced antioxidant capacity, reduced systemic inflammation, improved liver enzyme levels, insulin sensitivity, and anthropometric measures. This dietary intervention reduces MAFLD-related factors, which is promising in the context of disease management [87].

The beneficial effects of the MedDiet are partly attributed to its high content of monounsaturated fats, polyphenols, and fiber, bioactive compounds that will be discussed in detail in subsequent sections for their roles in oxidative stress reduction and insulin signaling.

### 4.3. DASH Diet

The DASH diet prioritizes reduced intake of sodium, saturated fats, and added sugars while emphasizing vegetables, fruits, lean meats, seafood, fish, and low-fat dairy products [88]. Typical macronutrient distribution for the DASH diet comprises approximately 18% protein, 27% fats, and 55% carbohydrates [89]. This dietary pattern consistently demonstrates beneficial effects on reducing blood pressure, body weight, fat mass, FBG, insulin levels, and leptin concentration [90]. Clinical studies in patients with MAFLD indicate that the DASH diet significantly lowers liver enzyme concentrations (alanine transaminase (ALT), alkaline phosphatase (ALP)), reduces IR, and enhances insulin sensitivity [91]. When integrated with time-restricted feeding (16:8), the DASH approach also significantly reduces hepatic steatosis, fibrosis scores, TGs, and anthropometric measurements (BMI, abdominal circumference (AC)) [92]. The DASH diet adherence effectively raises HDL-C levels [93] and reduces TCh, LDL-C, and VLDL, thereby improving cardiovascular risk profiles [94]. The DASH diet is an excellent choice for patients with MAFLD and associated conditions, such as hypercholesterolemia or type 2 diabetes (T2D), as it improves metabolic outcomes and reduces adipose tissue in the long term [91,92]. DASH also reduced liver enzymes and insulin resistance.

Given the role of inflammation in obesity pathophysiology [95], the DASH diet may positively affect immune function, including lymphocyte profile modulation in overweight and obese adults [96]. Additionally, women with PCOS adhering to DASH experienced improvements in reproductive hormone profiles, abdominal fat reduction, and improved insulin sensitivity [97,98].

Nutrients in the DASH diet include antioxidants, dietary fiber, magnesium, and calcium—factors known for their anti-inflammatory and antioxidant properties [99,100,101], which are beneficial to blood pressure and cardiovascular health.

### 4.4. Ketogenic and Low-Carbohydrate Diets

Ketogenic diets, characterized by high-fat, moderate protein, and very-low-carbohydrate intake, induce a state of ketosis, wherein the body primarily utilizes ketone bodies rather than glucose as fuel [102]. Lower carbohydrate intake leads to reduced insulin secretion, suppressed lipogenesis, and increased lipolysis, facilitating rapid weight and fat loss, particularly within the initial 12 weeks of implementation [102,103]. Significant reductions in body weight (often ~5–10%), WC, hip, and thigh circumferences (HC and TC) have been reported consistently [104]. Clinical trials demonstrate notable metabolic improvements, including decreased FBG, glycated hemoglobin (HbA1c) (down by ~1–2 percentage points in patients with diabetes), TGs, and increased HDL-C levels in overweight and obese individuals [105]. Due to beneficial outcomes on metabolism regulation and adipose tissue reduction, there is promise of long-term effects in improving MetS, cardiovascular outcomes, and diabetes management [106,107,108]. Beneficial effects on lipoprotein insulin resistance (LPIR), triglyceride-rich lipoprotein (TRL-P), adiponectin, and lipoprotein(a) (Lp(a)) have also been observed [108]. However, caution is warranted, as ketogenic diets may adversely impact lipid profiles in individuals with normal body weight or underlying predispositions [109,110]. Lower carb intake has also been associated with favorable outcomes on HDL-C and TGs concentration, HC, WC, weight [106,107,108] and body fat [107]. Ketogenic diets also show substantial benefits in improving hormonal balance, reducing IR, and enhancing lipid metabolism in women with PCOS, highlighting their efficacy in this subgroup [105,111]. In overweight or obese women with PCOS, ketogenic diets significantly improved reproductive hormones (dehydroepiandrosterone (DHEA), free testosterone, follicle-stimulating hormone (FSH), luteinizing hormone (LH)), lipid and glucose profiles, IR, and anthropometric indices. Fat mass, WC, and HC were all significantly reduced [112]. Regardless of the specific dietary approach, fat loss is central to the initial management of PCOS, as it improves hormonal balance by reducing androgen aromatization and improving the LH: FSH ratio [102].

A well-balanced ketogenic diet may be a great source of some bioactive compounds, including omega-3 fatty acids, contained in fish, oil, and nuts, as well as polyphenols and antioxidants found in low-carb fruits.

### 4.5. Plant-Based and Vegan Diet

Plant-based and vegan diets exclude animal-derived products, emphasize vegetables, legumes, fruits, whole grains, and nuts, and have attracted growing interest due to both ethical considerations and metabolic benefits [113,114]. They promote polyunsaturated fat intake and reduce trans and saturated fats. In contrast to omnivorous diets, vegan diets provide only plant-based protein sources, limiting intake of amino acids such as leucine and histidine, which may reduce IR in overweight individuals [115,116,117,118]. Similarly to DASH, plant-based diets are rich in antioxidants, contributing to reduced oxidative stress and improved insulin sensitivity [119]. In individuals with obesity, vegan diet adherence has led to improved lipid profiles, reductions in FBG, FBI, and ectopic fat deposits (additional—6.5 kg weight loss compared to the control group, −18.7 mg/dL drop in LDL cholesterol) [115,117]. Weight and fat mass loss have been associated with decreased leptin levels and improved appetite regulation [115,116,117,119]. However, these diets may lead to deficiencies in nutrients such as vitamin B12, iodine, and calcium if not properly managed [114]. Vegetarian diets are also associated with reduced systemic inflammation [120], improved lipid profiles, and comparable cardiovascular risk and body composition outcomes compared to omnivores [121]. Long-term epidemiological data also show ~20–25% reduced risk of type 2 diabetes among those adhering to predominantly plant-based patterns

The richness of dietary fiber and prebiotics supports insulin-glucose metabolism and gut microbiota. Dietary fiber and prebiotics, together with probiotics and postbiotics, improve metabolic health, immune signaling, and reduce inflammation.

### 4.6. Distinct Effects of Dietary Approaches on Metabolic Health and Long-Term Disease Prevention

Different dietary patterns exert unique effects on metabolic health through their influence on glucose regulation, lipid metabolism, inflammation, and body weight. The Mediterranean diet, rich in fruits, vegetables, whole grains, olive oil, and fish, has been extensively linked to improved insulin sensitivity, reduced inflammation, and better lipid profiles, contributing to lower risks of cardiovascular disease and type 2 diabetes. The DASH (Dietary Approaches to Stop Hypertension) diet emphasizes low sodium and high intake of fruits, vegetables, and low-fat dairy, effectively lowering blood pressure and improving lipid parameters.

Plant-based diets, especially whole-food vegetarian and vegan diets, are associated with lower BMI, improved glycemic control, and reduced inflammatory markers, primarily due to high fiber and antioxidant intake. However, potential nutrient deficiencies (e.g., B12, iron) may arise with poorly planned long-term adherence.

In contrast, the ketogenic diet, characterized by high fat and low carbohydrate intake, can rapidly improve glycemic control and induce weight loss, particularly in individuals with insulin resistance. However, concerns remain regarding its long-term cardiovascular impact due to elevated LDL levels and restricted intake of fiber and certain micronutrients.

Overall, diets emphasizing whole, minimally processed foods and rich in bioactive compounds (e.g., Mediterranean and DASH) offer the most consistent evidence for long-term metabolic benefits and chronic disease prevention. Tailoring dietary patterns to individual needs, metabolic profiles, and sustainability is essential for optimizing outcomes.

The key effects of the analyzed interventional and observational studies in individuals with overweight and obesity are summarized in Table 1 and Table 2.

## 5. Bioactive Compounds and Their Role in Metabolic Regulation

Dietary bioactive components, such as polyphenols, omega-3 fatty acids, dietary fibers, prebiotics, probiotics, postbiotics, vitamins, and microelements, are commonly recognized for their nutritional benefits. While dietary patterns provide an overall framework for metabolic regulation, their health effects are largely mediated by specific bioactive compounds found in foods. The following section explores how individual nutrients and phytochemicals influence molecular pathways relevant to metabolic health. There is growing interest in their potential beneficial effects on the pathophysiological mechanisms of obesity and metabolic disorders. These compounds present several mechanisms of action involved in health improvement, including antioxidants and anti-inflammatory activity, insulin metabolism regulation, and gut microbiome changes.

### 5.1. Polyphenols

Polyphenols are commonly found in plant-sourced foods [122]. These compounds are mostly known for their free radical scavenging properties. They suppress reactive oxygen species (ROS) generation, reduce oxidant enzymes, boost other antioxidant molecules, and act as chelating agents for metals [123]. Polyphenols also raise fatty acid oxidation and show a beneficial effect on glucose uptake. Polyphenols are also efficient in lowering lipid levels by hepatocellular AMP-activated protein kinase (AMPK) downregulation. Thus, they may help in hiperlipidemia and atherosclerosis improvement [124]. Since disordered glucose metabolism may lead to oxidative stress [125], their function in energy and glucose metabolism appears crucial. Oxidative stress is strongly correlated with chronic inflammation and a greater risk of cardiometabolic complications, which all occur with obesity [126]. Polyphenols raise fat cell apoptosis and reduce adipogenesis and lipogenesis [127]. They also reduce levels of cytokines including IL-1β, and nitric oxide (NO) [128]; along with modulating obesity-related molecular pathways, leading to adipose reduction and anti-inflammatory effects by decreasing TNF-α-induced (nuclear factor kappa B) NF-κB activation, subsequently promoting an increase in anti-inflammatory agents [128,129]. These compounds regulate glucagon-like peptide (GLP)-1 levels, suppress liver gluconeogenesis and glucagon secretion, improving insulin-glucose metabolism, insulin sensitivity, IR, (fasting glucose decrease and drop in HOMA value with resveratrol supplementation), lowering LDL cholesterol by ~5–10% in controlled trials and reducing postprandial hunger [130]. Additionally, polyphenols improve intestinal homeostasis and inflammation, leading to short-chain fatty acids (SCFAs) production. They promote beneficial gut microbiota [131] and suppress pathogenic microbes, leading to demonstrable benefits in cardiometabolic health and cancer prevention [122].

### 5.2. Omega-3 Fatty Acids

Omega-3 fatty acids are a class of polyunsaturated fatty acids (PUFAs) [132] that are predominantly found in oily fish [133]. They have demonstrated significant cardiovascular benefits, particularly in high-risk populations, through mechanisms such as the stabilization of atherosclerotic plaques, which may reduce the incidence of cardiovascular events and related mortality [134]. Additionally, omega-3 fatty acids play a preventive role in the development of hypertriglyceridemia and hyperlipidemia by decreasing de novo lipogenesis, reducing hepatic secretion of lipoproteins full of TGs, as well as by apoB degradation due to lower amounts of TGs [135]. Furthermore, PUFAs, particularly omega-3 and omega-6 fatty acids, play a crucial role in lipid metabolism by modulating gene expression and enzyme activity involved in lipid regulation. PUFAs activate peroxisome proliferator-activated receptors (PPARs), which enhance fatty acid oxidation and reduce hepatic lipogenesis. Additionally, they help lower plasma triglyceride levels, improve HDL cholesterol, and reduce LDL cholesterol through mechanisms involving altered lipoprotein synthesis and clearance. These effects contribute significantly to the prevention and management of dyslipidemia and other metabolic disorders. Clinically, omega-3 supplementation (or oily fish intake) helps stabilize atherosclerotic plaques and improve insulin sensitivity by reducing ectopic fat accumulation.

Similarly to polyphenols, omega-3 fatty acids stimulate the secretion of GLP-1 in the gastrointestinal tract with beneficial effects on satiety. This promotes increased insulin secretion, improved insulin sensitivity, and a reduction in FBG levels. They further enhance insulin action by modulating adipokine levels and reducing adiposity [136]. Moreover, omega-3 fatty acids help prevent IR by mitigating endoplasmic reticulum stress and enhancing mitochondrial β-oxidation of fatty acids [137]. Increased β-oxidation contributes to reduced lipogenesis and lipid accumulation, thereby supporting improved insulin sensitivity [138]. Omega-3 fatty acids also function as antioxidants by scavenging superoxide and reducing ROS production [139]. Their anti-inflammatory effects are mediated through modifications to cell membrane lipid composition [68], inhibition of pro-inflammatory cytokine production [140] and upregulation of anti-inflammatory mediators [133]. Additionally, omega-3 fatty acids inhibit leukocyte activation and recruitment, thereby promoting the resolution of inflammation and attenuating oxidative stress [133,141]. Furthermore, omega-3 fatty acids contribute to gut health by reducing endotoxin levels and suppressing pro-inflammatory mediators, while enhancing anti-inflammatory compounds. These effects support the maintenance of gut microbiota balance and intestinal homeostasis regulation [142,143].

### 5.3. Dietary Fibers and Prebiotics

Dietary fibers and prebiotics, primarily found in plant-based foods, support gut and cardiovascular health [144,145]. They are metabolized into SCFAs, which enhance metabolism, reduce lipogenesis, improve lipid profiles, and also benefit gut barrier function [146,147,148]. Prebiotics are “non-digestible nutritional ingredients that beneficially affect the host, selectively stimulating the growth and activity of one or more beneficial bacteria in the colon, improving the health of its host” [149]. The agents are substrates which are fermented by probiotics that nourish beneficial gut bacteria, decrease inflammation, and reduce insulin secretion, improving IR [150,151]. Fiber intake slows gastric emptying, enhances satiety, and delays nutrient, including glucose, absorption, leading to improvement of lipid profiles, better insulin sensitivity, and body weight reduction [152]. Their health effects are amplified by polyphenols, and their anti-inflammatory and antioxidant properties [151,153].

### 5.4. Probiotics and Postbiotics

Probiotics are live microorganisms, including *Lactobacillus*, *Bifidobacterium*, and *Saccharomyces boulardii*, commonly found in fermented dairy products and pickled foods [154,155]. These beneficial microbes exert positive effects on host health primarily through the modulation of gut microbiota composition and the production of lactic acid, which is subsequently converted into metabolically active SCFAs [156]. SCFAs play a key role in enhancing the secretion of GLP-1, thereby contributing to improved insulin-glucose metabolism, similar to the effects observed with polyphenols, and omega-3 fatty acids [157].

In addition, probiotics influence immune signaling by modulating NF-κB pathways, suppressing expression of pro-inflammatory cytokines [158] and improving antioxidant defenses through the upregulation of free radical scavenging activity [159]. Postbiotics, the bioactive metabolic by-products of probiotics, act as mediators of these beneficial effects. They mimic the health-promoting properties of probiotics and provide a safer alternative due to their enhanced stability, longer shelf life, and lack of live organisms, thus avoiding risks associated with live microbial intake. In comparison to probiotics, postbiotics use allows avoidance of allergic sensitization, opportunistic infections, and autoimmune disorders, which is crucial for newborns and high-risk patients. Postbiotics have gained increasing attention as a functional component of microbiome-targeted therapies [160,161,162]. Postbiotics such as short-chain fatty acids (SCFAs) exert anti-inflammatory, immunomodulatory, and metabolic benefits.

### 5.5. Vitamin D

Vitamin D deficiency is an escalating public health concern and is significantly associated with an increased risk of developing metabolic disorders, including osteoporosis, autoimmune diseases, cardiovascular events, and T2D [163]. Low serum vitamin D levels are more frequently observed in individuals with obesity. Additionally, vitamin D is involved in adipose tissue regulation and metabolism, and its deficiency is strongly correlated with excess adiposity [164]. Obese people present elevated storage of vitamin D in adipose tissue and disruption of vitamin D binding proteins [165]. Moreover, this group of patients demonstrates worse metabolic outcomes of vitamin D supplementation compared to patients with lower BMI [166]. Vitamin D insufficiency may downregulate insulin receptor expression and increase inflammation, contributing to the development of IR [167]. Adequate vitamin D status supports insulin signaling and enhances the activity of transcription factors involved in fatty acid and adipose tissue metabolism, thereby improving insulin sensitivity and promoting the reversal of IR [168,169]. Additionally, vitamin D plays a protective role against mitochondrial dysfunction. It reduces lipid peroxidation and improves total antioxidant capacity (TAC) by stimulating the production of antioxidant enzymes and molecules [170]. By decreasing ROS generation in adipose tissue, vitamin D acts as a metabolic antioxidant [171]. Beyond its antioxidant properties, vitamin D increases the expression of anti-inflammatory cytokines and suppresses pro-inflammatory cytokines and signaling pathways [172]. In the gastrointestinal tract, it mitigates local inflammation and supports microbial integrity, promoting a diverse and balanced gut microbiota while reducing pathogenic bacterial populations [173].

### 5.6. Magnesium

Magnesium plays a fundamental role in numerous biological processes [174]. As a cofactor in many enzymatic reactions, it is essential for proper insulin signaling [175]. It also upregulates glucose transporter-4 (GLUT-4) gene expression, enhancing glucose uptake in peripheral tissues and reducing IR [176]. Magnesium supports mitochondrial function and reduces ROS production, earning recognition as a mitochondrial antioxidant [177]. Magnesium deficiency exacerbates existing oxidative stress by increasing ROS generation and diminishing antioxidant defenses in obese individuals. Additionally, hypomagnesemia contributes to inflammation by stimulating pro-inflammatory cytokines and suppressing anti-inflammatory mediators [178]. This pro-inflammatory and oxidative environment favors the development of MetS and increases the risk of cardiovascular complications [179,180]. Magnesium deficiency also negatively affects intestinal health, potentially reducing beneficial microbial populations and increasing inflammation [181]. Supplementation has been shown to improve gut microbiota composition and elevate SCFAs levels, thereby enhancing gut barrier integrity, regulating lipid metabolism, and reducing oxidative stress and inflammation [182,183].

The key mechanism of action and effects of the bioactive compounds are summarized in Table 3.

## 6. Advances in Metabolomics and Nutritional Biomarker Discovery

In the context of obesity, personalized nutrition is gaining increasing attention due to individual differences in biochemical patterns, metabolic pathways, and gut microbiota composition. Standardized dietary approaches often fail to produce consistent outcomes, prompting clinicians to explore tailored nutritional strategies. Metabolomics, a field that analyzes small-molecule metabolites in tissues, cells, and bodily fluids; offers valuable insights [176,177] into the real-time metabolic state of an individual. Unlike transcriptomics or proteomics, metabolomics provides direct data on downstream products of metabolic processes, enabling precise assessment of both deficiencies and excesses in micro- and macronutrients [184]. Advanced diagnostic techniques include nuclear magnetic resonance (NMR), mass spectrometry (MS), gas chromatography-MS (GC-MS), liquid chromatography-MS (LC-MS), capillary electrophoresis (CE), [185]. Additionally, Fourier transform infrared spectroscopy (FT-IR) and Raman spectroscopy have enhanced our capacity to investigate metabolic changes and disease risk markers [186].

These molecular insights offer the foundation for emerging personalized dietary strategies, as outlined below.

### 6.1. Role of Metabolomics in Detecting Subtle Metabolic Changes

Metabolomics allows for the detection of metabolic alterations prior to clinical manifestation. By analyzing hundreds of compounds simultaneously in biological samples like blood, urine, or saliva, this approach can identify subclinical changes that are often missed by traditional lab tests.

Badoud et al. [187] examined the metabolic response to a high-calorie meal in lean controls, MHO, and MUO individuals. MHO participants exhibited a glucose-insulin profile similar to lean controls, whereas the MUO group showed significantly elevated postprandial glucose and insulin levels. Lower concentrations of PUFAs in the MHO group indicated preserved metabolic flexibility. Additionally, a strong correlation was found between BCAAs, saturated fatty acids, and glucose-insulin responses, suggesting their potential as early markers of metabolic dysfunction.

In another review, over 300 metabolites, especially lipids, aromatic amino acids, BCAAs, and carbohydrate derivatives, were identified as predictive biomarkers of T2D. Alterations in their levels often precede overt hyperglycemia, allowing for early risk assessment and preventive strategies [188]. A non-targeted LC-MS study comparing women with morbid obesity (MO) to those with a healthy weight found elevated serum choline and acylglycerols, as well as reduced levels of bile acids, steroids, ceramides, and phospholipids in the MO group. In those with both MO and T2D, additional metabolic derangements were observed, underscoring the potential of metabolomics in clinical stratification [189].

### 6.2. Metabolomics in Dietary Intervention Research

Metabolomics has become essential in evaluating the impact of dietary interventions. It enables the identification of food intake biomarkers and the monitoring of physiological responses to dietary changes in both observational and randomized controlled trials (RCTs).

For example, Tang et al. [189,190] used targeted LC-MS to detect urinary metabolites such as ferulic acid and quercetin following consumption of whole grains, fruits, and vegetables. Zhu et al. [191] identified alkylresorcinol metabolites as reliable biomarkers of short-term whole grain intake.

Beyond tracking dietary components, metabolomics allows assessment of individual metabolic responses to dietary interventions, facilitating the concept of “metabotyping”—classifying individuals by their metabolic phenotype to optimize diet efficacy. Pigsborg and Magkos [191] highlighted that specific metabotypes may predict differential responses in insulin sensitivity, TGs levels, and inflammatory markers.

Garcia-Pérez et al. [192,193] conducted a crossover trial in which 19 healthy individuals followed four diets varying in compliance with World Health Organization (WHO) dietary guidelines. Metabolomic analysis of urine identified 19 biomarkers linked to high adherence, including hippurate and methylhistidines. In contrast, low-compliance diets were associated with elevated levels of carnitine, creatine, and glucose. These findings emphasize metabolomics’ utility in linking dietary patterns to biological outcomes.

As noted by Beckmann et al. [194], real-time biomarker tracking in urine enables early detection of metabolic changes, supporting the proactive management of at-risk populations.

Summing up, using metabolomics in RCTs is a groundbreaking approach to dietetics and personalized nutrition. They enable not only the identification of metabolites characteristic of the consumption of specific foods, but also the assessment of the effectiveness of the diet on the metabolic level, personalization of recommendations and monitoring of therapeutic effects. The use of metabolomics in RCTs is therefore a direction to explore in light of the future of precision nutrition and treatment of metabolic diseases.

### 6.3. Personalized Nutrition Approaches

Modern dietetics is moving beyond universal dietary guidelines toward individualized plans informed by genetic, epigenetic, microbiota, and metabolomic profiles. Precision nutrition leverages metabolomics to assess dietary status and monitor responses to interventions.

Two key concepts, nutritypes and metabolotypes, aid in personalizing dietary interventions. Nutritypes reflect long-term biochemical adaptations to diet, while metabolotypes classify individuals based on acute metabolic responses [195]. This differentiation is particularly crucial in obesity, where individuals with similar BMI may exhibit divergent metabolic phenotypes.

Brennan and de Roos [195] emphasized that metabolomic profiles can help tailor dietary recommendations by predicting response to high-fiber or low-fat diets. Integrating metabolomics with DNA methylation, miRNA expression, and gut microbiota data allows for a more nuanced prediction of dietary outcomes.

Genetic data alone is insufficient due to the complex interplay of genes with environmental and nutritional factors. Voruganti [196] illustrated how combined gene variants, epigenetics, and microbial metabolites affect energy homeostasis, fat accumulation, and nutrient needs. This integrative approach facilitates the development of customized nutritional strategies based on an individual’s unique “metabolic footprint”.

LeVatte et al. [197] underscored the role of metabolomics in identifying dietary biomarkers, detecting micronutrient imbalances, and evaluating intervention effectiveness. Van Ommen et al. [198] proposed a systems biology model where “systemic flexibility,” the body’s ability to maintain homeostasis after stimuli such as meals, serves as a cornerstone for dynamic and adaptive dietary planning.

Significant interindividual differences in metabolic biomarker responses, identified through metabolomics, allow clinicians to recognize patients who respond poorly to specific dietary interventions. As mentioned earlier, a key role in this variability is played by genetics, epigenetics, and gut microbiota, all of which contribute to the composition of a patient’s metabolome. Identifying how an individual’s metabolome responds to specific micro- and macronutrients can serve as a blueprint for diet composition, indicating which nutrients should be included or excluded from the patient’s daily eating habits.

Metabolomics, by revealing both long-term adaptation to diet and short-term reactions to nutrients, can help predict who would benefit from a particular dietary intervention, before introducing it, therefore enhancing the process of customizing nutrition. Moreover, analyzing metabolite levels associated with diet composition allows us dynamic reaction to any deficiencies or surpluses as well as assessment of the effectiveness of our interventions. Including data on DNA methylation, miRNA expression, and microbiota additionally improves those predictions and enables dynamic adjustment of recommendations over time.

Human intervention studies combined with metabolomics, transcriptomics, and microbiome profiling can reveal how these compounds affect gene expression, enzyme activity, gut microbial composition, and metabolite production. For example, polyphenols may enhance insulin sensitivity by modulating inflammatory gene expression and improving mitochondrial function, while probiotics influence metabolic outcomes by altering short-chain fatty acid production and gut barrier integrity.

Population-specific factors—such as genetics, baseline microbiota composition, diet, age, and lifestyle—significantly influence these responses. Therefore, stratified or personalized trials, considering ethnic, genetic, and microbiome variability, are essential to clarify inter-individual differences in response to bioactives.

Moreover, in vitro and in vivo mechanistic studies, including cell culture and animal models using humanized microbiota, can further elucidate the molecular mechanisms underlying these effects.

### 6.4. Integrating Environmental, Microbiological, and Genetic Factors into Personalized Nutrition for Metabolic Health

Personalized nutrition strategies can be significantly enhanced by incorporating environmental, microbiological, and genetic factors. Genomic and epigenetic profiling helps identify individual susceptibilities to metabolic disorders (e.g., variants in genes related to lipid metabolism or insulin sensitivity), enabling nutrient-gene interaction analysis (nutrigenetics). Gut microbiome sequencing (e.g., 16S rRNA or metagenomic analysis) allows classification of microbial diversity and identification of dysbiosis patterns linked to obesity, insulin resistance, and inflammation, guiding interventions such as prebiotic, probiotic, or fiber-rich diets.

Environmental factors, including physical activity, stress, sleep, exposure to pollutants, and socioeconomic context, can be integrated using wearable technologies, digital health tools, and behavioral assessments. These data streams, when combined through multi-omics platforms and machine learning algorithms, facilitate dynamic and adaptive dietary recommendations tailored to individual physiology and lifestyle.

Together, these approaches enable a systems biology model of personalized nutrition, targeting the underlying causes of metabolic dysfunction and supporting long-term management of metabolic disorders.

## 7. Dietary Supplements and Functional Foods

Dietary supplements are food-like products designed to complement the normal diet, typically consisting of concentrated sources of vitamins, minerals, or other substances with nutritional or physiological effects. They are commonly available in forms such as tablets, capsules, powders, or liquids [199,200]. Unlike pharmaceuticals, supplements are not intended to treat or prevent diseases but may support physiological functions and contribute to restoring homeostasis. Functional foods, though lacking a universally accepted definition, refer to food products enriched with bioactive compounds such as dietary fiber, phytosterols, omega-3 fatty acids, polyphenols, or probiotics, which exert health-promoting effects that extend beyond basic nutrition [200,201,202,203]. The relevance of both supplements and functional foods is increasing amid the global rise in metabolic diseases. Current estimates suggest that one in four individuals worldwide meets the criteria for MetS, with many experiencing nutrient deficiencies despite excessive caloric intake [199,204]. As adjuncts to pharmacological and dietary interventions, dietary supplements and functional foods can play an essential role in the prevention and management of metabolic complications. Bioactive substances in these products may support glucose and lipid metabolism, mitigate oxidative stress, enhance insulin sensitivity, and improve gut microbiota composition [199,201,204,205]. However, their effectiveness depends on factors such as the bioavailability of active compounds, the method of administration, health status, dietary patterns, and interactions with medications or other nutrients [200,201]. In recent literature, functional food components have been increasingly evaluated not only for their biological properties but also for their role in preventing specific chronic diseases. Examples include probiotic-rich fermented milk, plant sterol-fortified spreads, and flavonoid-enriched juices, which are now considered integral tools in the dietary management of obesity and IR [203,204,205]. The efficacy of such products is progressively supported by population-based and clinical research [201,202,205].

### 7.1. Supplements in the Context of Poor-Quality Diets

Individuals with obesity frequently consume diets high in processed foods, characterized by excessive caloric density and low nutrient value. Studies reveal common deficiencies in micronutrients such as iron, zinc, selenium, magnesium, vitamin D, B-group vitamins, and antioxidants (e.g., vitamins C and E) within this population [206,207,208]. Chronic inflammation and increased adiposity can further impair the absorption of these nutrients, exacerbating deficiencies and contributing to elevated oxidative stress and impaired insulin-signaling factors that promote the development of MetS and its comorbidities [166,206]. For instance, vitamin D deficiency is observed in up to 70–90% of individuals with obesity [166,209], Supplementation with vitamin D has been shown to improve glucose metabolism, reduce insulin concentrations, and mitigate systemic inflammation [209,210]. This is particularly relevant during autumn and winter months when cutaneous synthesis is significantly reduced. Magnesium supplementation has also demonstrated benefits, including enhanced endothelial function, improved insulin sensitivity, and blood pressure reduction [208,210]. Given its critical role in mitochondrial function, magnesium deficiency may aggravate metabolic dysfunction in individuals with excess adiposity. Similarly, omega-3 fatty acid supplementation has been linked to improvements in lipid profiles, reduced IR, and anti-inflammatory effects by modulating membrane composition and gene expression involved in metabolic regulation [211,212].

Although supplements do not replace a balanced diet, they serve as valuable adjuncts, particularly in high-risk groups. Studies have shown that complex supplement formulations (e.g., combining vitamin D, B12, magnesium, and omega-3 fatty acids) can improve clinical markers such as HOMA-IR, TGs, and inflammatory indices [166,207,213]. Notably, synergistic effects have been observed, such as co-supplementation of vitamin D and magnesium yielding greater improvements than either agent alone [210]. Additionally, microbiota-targeted interventions, including probiotics and prebiotics, may support micronutrient absorption, reinforce gut barrier integrity, reduce endotoxemia, and enhance metabolic regulation [207].

Emerging literature emphasizes that correcting micronutrient deficiencies should be viewed not only as nutritional supplementation but as a therapeutic strategy in metabolic disease management. However, this approach must be individualized and supervised to prevent potential adverse interactions or excessive intake.

### 7.2. Regulation and Safety

Despite their therapeutic potential, dietary supplements are legally classified as food products rather than medications. In the European Union, they are governed by Directive 2002/46/EC, which defines supplements as products containing concentrated sources of nutrients or other substances with nutritional or physiological effects [214]. These products must comply with standards concerning quality, purity, and labeling, and are assessed by the European Food Safety Authority (EFSA) for safety and maximum allowable levels [214,215,216]. In the United States, dietary supplements fall under the Dietary Supplement Health and Education Act (DSHEA) of 1994, which places responsibility for product safety on the manufacturer. The Food and Drug Administration (FDA) does not require premarket approval but may intervene post-marketing if safety issues arise [217]. This system has drawn criticism for allowing low-quality or adulterated products to enter the market. Independent analysts estimate that up to 20% of supplements in the U.S. contain impurities or composition discrepancies [218,219]. Safety concerns are particularly relevant with long-term use, high-dose intake, or unsupervised combinations of multiple supplements. Reported adverse effects include hepatotoxicity from certain plant extracts, hypercalcemia from excessive vitamin D, or sympathomimetic effects from caffeine-containing products [215,220]. Contamination has also led to positive doping tests among athletes, underscoring the need for rigorous quality control [221]. While European member states (e.g., Poland) require supplement notification to health authorities (such as the Chief Sanitary Inspectorate), mandatory certification and clinical efficacy data are not required. This regulatory gap has led to calls for enhanced monitoring systems, including public databases that report testing results for purity and safety [214,215]. Regulatory bodies such as the EFSA and FDA are increasingly scrutinizing health claims associated with supplements and taking legal action against misleading advertising practices [214,217,222]. In clinical practice, supplement use should be evidence-based, tailored to individual needs, and conducted under professional guidance to minimize risks of inefficacy or harm.

### 7.3. Efficacy of Supplements in Clinical Trials

A growing body of clinical trials has explored the effectiveness of dietary supplements in modulating metabolic parameters among individuals with overweight, obesity, and MetS. While outcomes vary, meta-analyses support the efficacy of certain ingredients in improving glycemic control, lipid profiles, blood pressure, and inflammatory biomarkers [199,214,223].

Vitamin D supplementation, particularly in deficient individuals, has been shown to enhance insulin sensitivity, reduce FBG and insulin levels, and attenuate inflammation [223,224].

A 2024 meta-analysis by Oliveira et al. [223] reported significant improvements in glucose regulation, lipid levels, and blood pressure following vitamin D supplementation in diverse populations across Brazil, the U.S., and Europe. Long-term supplementation with vitamin D and omega-3 fatty acids was associated with preservation of leukocyte telomere length, a biomarker of aging and metabolic stress [224]. Omega-3 fatty acids—particularly eicosapentaenoic acid (EPA) and docosahexaenoic acid (DHA)—have shown benefits in lowering TGs, improving HDL-C/LDL-C ratios, and reducing inflammatory markers such as CRP and IL-6 [225]. These effects are most pronounced in individuals with established MetS or elevated cardiovascular risk.

Combined supplementation with vitamin D and magnesium has demonstrated synergistic effects, enhancing vitamin D activation, lowering blood pressure, and reducing inflammation [208,210,226]. Magnesium supplementation alone has also been linked to reductions in total cholesterol and LDL-C, especially at doses of 300–400 mg/day [210,227]. Other promising interventions include probiotics and prebiotics, which influence the gut–brain axis and modulate metabolic responses through SCFAs production. RCTs involving strains like *Lactobacillus rhamnosus*, *Bifidobacterium breve*, and *Saccharomyces boulardii* have reported improvements in insulin sensitivity, BMI reduction, and glycemic control in individuals with MetS. Prebiotics also improve mineral bioavailability and promote gut integrity [203,228].

Polyphenolic compounds (e.g., curcumin, resveratrol) and plant-based extracts (e.g., green tea) have demonstrated modest benefits in weight management, lipid modulation, and glycemic regulation [199,213]. However, their clinical efficacy is highly dependent on dosage, formulation, and bioavailability [213].

Despite encouraging findings, heterogeneity in study populations, supplement formulations, and duration limits generalizability [199,213]. Thus, future research should prioritize well-designed, long-term RCTs and consider integrating personalized supplementation based on individual metabolic profiles [199].

### 7.4. Supplements vs. Functional Foods: Which Is More Effective?

Both supplements and functional foods offer potential benefits in supporting metabolic health, but their relative effectiveness is context-dependent. Supplements provide standardized doses of active compounds, which are particularly beneficial in correcting identified deficiencies or delivering therapeutic doses rapidly. Functional foods, in contrast, integrate bioactive substances into the daily diet, offering a more natural and potentially synergistic approach to disease prevention and management. Clinical studies suggest that functional foods may match or surpass supplements in long-term efficacy for improving lipid profiles, glycemic control, and inflammatory markers [199,227,228]. Examples include probiotic-enriched yogurts, phytosterol-fortified spreads, and flavonoid-containing beverages—all of which have demonstrated favorable effects in MetS populations [199,202]. Functional foods also tend to promote better adherence, as they are consumed as part of regular meals and do not require additional supplementation routines. However, supplements offer more precise dosing and are indispensable in cases of severe deficiencies, rapid metabolic deterioration, or clinical settings such as bariatric surgery [208,213,224]. Bioavailability differences are also critical. Nutrients in whole foods may be better absorbed due to the presence of supportive cofactors (e.g., fats enhancing fat-soluble vitamin absorption). Furthermore, functional foods often deliver a combination of compounds (e.g., fiber, antioxidants, phytosterols) that can act synergistically to promote metabolic health [199,229]. Current dietary strategies advocate for a combined approach, leveraging both functional foods and targeted supplementation, to maximize therapeutic outcomes. This integrated approach, when combined with lifestyle modifications such as physical activity and stress reduction, offers a comprehensive strategy for managing obesity and MetS [203,229,230].

## 8. Challenges and Future Directions

Although the various diet adherence impacts on metabolic health are widely known, there are new challenges in the approach to therapeutic interventions. Inter-individual variability, including diversity in biochemical patterns, metabolic pathways and microbiota, is the reason for varying responses to the same dietary strategies. This necessitates the development of tailored nutrition. However, there is insufficient medical data, limited by short-duration studies, small sample sizes, heterogeneity of analyzed populations, and the type of nutritional interventions, making it difficult to generalize findings to a greater population. More comprehensive long-term mechanistic studies are needed to identify molecular mechanisms of the beneficial changes occurring as a result of implementing nutritional patterns.

Utilization of omics technologies provides an opportunity to generate detailed data on individual metabolic profiles. Metabolomics can be used to identify and quantify markers of metabolic health and assess physiological responses to dietary changes. Leveraging these technologies could facilitate improved metabolic health results due to targeted dietary approaches based on specific “metabolic footprint”.

## 9. Conclusions

Metabolic health is shaped by complex interactions between dietary patterns, bioactive food components, and individual genetic and lifestyle factors. This review highlights that evidence-based diets—particularly the Mediterranean, DASH, plant-based, and low-carbohydrate/ketogenic approaches—confer significant improvements in body weight, glycemic control, blood pressure, and lipid profiles. These benefits are largely mediated by specific nutrients and phytochemicals, unsaturated fatty acids, fibers, polyphenols, and probiotics act synergistically to enhance insulin sensitivity, stimulate GLP-1 secretion (improving satiety and glucose homeostasis), attenuate inflammation, and reduce oxidative stress. Notably, while ketogenic diets yield rapid short-term improvements (in obesity and polycystic ovary syndrome by reducing insulin resistance and visceral fat), their long-term cardiovascular effects require further study. Overall, diets rich in unprocessed plant foods, omega-3 fats, and antioxidant compounds emerge as the most consistently beneficial for long-term metabolic health and chronic disease prevention.

Looking ahead, the inter-individual variability in diet response calls for a personalized nutrition approach. Future research should leverage genomics, metabolomics, and gut microbiome profiling to tailor dietary interventions to an individual’s metabolic “fingerprint.” Such precision nutrition strategies could maximize therapeutic outcomes by predicting who will respond best to a given diet or bioactive supplement. High-quality, long-duration trials are needed to unravel the molecular mechanisms by which these nutritional interventions exert their effects and to confirm sustained benefits on hard clinical endpoints (diabetes incidence or cardiovascular events). Optimizing dietary patterns—possibly in combination with functional food supplementation—offers a powerful, non-pharmacologic tool to combat obesity-related metabolic disorders. Implementing these insights into clinical practice and public health policy, while accounting for personal differences, holds great promise for improving global metabolic health in the years to come.

## Figures and Tables

**Table 1:** Effects of the analyzed interventional studies in individuals with overweight and obesity.

Author (Year)	Type of Intervention/Diet	Type of Study	Sample Size (n)	Duration	Mean Age (Years)	Baseline BMI (kg/m^2^)	Anthropometric Outcomes	Glycemic & Metabolic Outcomes	Blood Pressure/Lipid Profile/Inflammation
Di Daniele N et al., (2013) [80]	Mediterranean Diet in Men vs. Mediterranean Diet in women	Interventional study	n = 25 n = 34	6 month	48.7 ± 13.0 51.4 ± 11.5	35.8 ± 4.3 40.6 ± 7.8	↓BMI *p* < 0.001–3.47; ↓body mass *p* < 0.001; −10.2 ↓fat mass *p* < 0.001; ↓WC *p* < 0.001; −19.81	↓FBG *p* < 0.001; −12.95	↓SBP *p* < 0.001; −11.48 ↓DBP *p* < 0.001; −9.69 ↓TCh *p* < 0.001; −25.52 ↓LDL-C *p* < 0.001; −9.98 ↓TG *p* < 0.001; −38.79
Asoudeh F et al., (2023) [84]	Mediterranean Diet vs. Control group	Interventional study	n = 35 n = 35	12 week	14 ± 1.0 14 ± 1.0	27 ± 3.9 28 ± .8	↓BMI *p* < 0.001; −1.1 ↓body mass *p* < 0.001; −2.8 ↓WC *p* < 0.001; −2.9	↓FBG *p* < 0.001; −6.7 ↓HOMA-IR *p* < 0.001; −1.1	↓SBP *p* < 0.001; −9.0 ↓LDL-C *p* < 0.001; −15.1 ↑HDL-C *p* < 0.001; +3.7 ↓TG *p* < 0.001; −29.7 ↓hs-CRP *p* = 0.02; −1.4 ↓IL-6 *p* = 0.02; −31.7
Yurtdaş G et al., (2022) [87]	Mediterranean Diet vs. Control group	Interventional study	n = 22 n = 22	12 week	13.0 ± 2.0 13.9 ± 2.3	30.9 ± 5.2 33.7 ± 5.5	↓BMI *p* < 0.001; −2.1 ↓body mass *p* < 0.001; −5.1 ↓fat mass (%) *p* < 0.001; −3.9 ↓WC *p* < 0.001; −8.1 ↓HC *p* < 0.001; −5.5	↓insulin *p* = 0.01; −1.4 ↓HOMA-IR *p* = 0.02; −0.5 ↓AST *p* < 0.001; −10.0 ↓ALT *p* < 0.001; −18 ↓GGT *p* < 0.001; −6.0	↓CRP *p* = 0.008; −0.2
Rodríguez-López CP et al., (2021) [96]	DASH diet in normal BMI participants vs. DASH diet in overweight vs. DASH diet in obese participants	Interventional study	n = 29 n = 14 n = 16	8 week	25.9 ± 6.5 for the whole group	Normal 21.3 ± 2.1 Overweight 26.8 Obese 31.9	↓body mass *p* < 0.001; −2.0 ↓fat mass *p* < 0.001; −1.1 ↓VAT *p* < 0.02; −4.0 ↓WC *p* < 0.001; −5.4	-	-
Kahleova H et al., (2020) [115]	Vegan diet vs. Control group	Interventional study	n = 122 n = 122	16 week	53.0 ± 10.0 57.0 ± 13.0	33.3 33.6	↓BMI *p* < 0.001; −1.9 ↓body mass *p* < 0.001; −6.4 ↓fat mass *p* < 0.001; −4.1	↓FBG *p* = 0.001; −0.6 ↓FBI *p* = 0.006; −46.2 ↓HOMA-IR *p* < 0.001; −1.3 ↑PREDIM *p* < 0.001; +0.9	↓TC *p* < 0.001; −0.6 ↓LDL-C *p* < 0.001; −0.4
Kahleova H et al., (2019) [116]	Vegan diet vs. Control group	Interventional study	n = 38 n = 37	16 week	53.2 ± 12.6 for the whole group	28 to 40 kg/m for the whole group	↓BMI < 0.001; −2.0 ↓body mass *p* < 0.001; −6.5 ↓fat mass *p* < 0.001; −4.3	↓HOMA-IR *p* = 0.004; −1.0	-
Kahleova H et al., (2018) [117]	Vegan diet vs. Control group	Interventional study	n = 38 n = 37	16 week	52.6 ± 14.7 54.3 ± 9.9	33.1 33.6	↓BMI < 0.001; −2.0 ↓body mass *p* < 0.001; −6.5 ↓fat mass < 0.001; −4.3 ↓VAT < 0.001; −224	↓FBG *p* < 0.001; −0.4 ↓FBI < *p* = 0.05; −85.4 ↓HOMA-IR *p* = 0.004; −1.0	↓TCh *p* = 0.02; −1.1 ↓LDL-C *p* = 0.03; −0.9
Sun J et al., (2023) [107]	Low-carbohydrate diet vs. Calorie-restricted diet vs. Low-carbohydrate and calorie-restricted diet vs. Control group	Interventional study	n = 76 n = 72 n = 76 n = 74	12 week	34.2 ± 7.8 33.6 ± 6.4 33.2 ± 7.0 35.1 ± 8.2	30.2 ± 3.8 30.3 ± 3.4 31.0 ± 4.7 29.9 ± 3.9	↓BMI *p* < 0.001; −2.3 ↓body mass *p* < 0.001; −5.9 ↓fat mass (%) *p* < 0.001; −2.5 ↓WC *p* < 0.001; −5.5	-	-
Velázquez-López L et al., (2014) [85]	Mediterranean diet vs. Control group	Interventional study	n = 24 n = 25	16 week	11.2 ± 2.7 11.4 ± 2.9	27.3 ± 3.9 26.7 ± 4.7	↓BMI *p* = 0.001; −1.1 ↓fat mass *p* < 0.001; −2.6 ↓lean mass *p* < 0.001; 2.1	↓glucose *p* < 0.001; −10.5	↓TCh *p* < 0.001; −31.0 ↓LDL-C *p* < 0.001; −22.0 ↑ HDL-C *p* < 0.001; +9.0 ↓TG *p* < 0.001; −90.0
Michalczyk MM et al., (2020) [111]	Ketogenic diet vs. Control group	Interventional study	n = 46 n = 45	12 week	42 ± 7.0 41 ± 6.0	32.5 ± 4.5 33.2± 4.6	↓body mass *p* = 0.001; −13.72 ↓WC *p* = 0.001; −13.7 ↓HC *p* = 0.001; 11.61 ↓TC *p* = 0.001; −7.66	↓FBG *p* = 0.001;−2.2 ↓insulin *p* = 0.001; −10.51 ↓HbA1c *p* = 0.002; −0.5 ↓HOMA-IR *p* = 0.001; −2.35	↑HDL-C *p* = 0.001; + 16.28 ↓TG *p* = 0.001; −84.2
Asemi Z et al., (2015) [98]	DASH diet vs. Control group	Interventional study	n = 24 n = 24	8 week	30.7 ± 6.7 29.4 ± 6.2	29.1 ± 3.2 31.5 ± 5.7	↓WC *p* = 0.003; −5.2 ↓HC *p* < 0.0001; −5.9	↓insulin *p* = 0.03; −1.88 ↓HOMA-IR *p* = 0.01; −0.45	↓hs-CRP *p* = 0.009; −763.29
Kucharska A et al., (2018) [90]	DASH diet vs. Control group	Interventional study	n = 64 n = 62	12 week	61.3 ± 7.9 58.1± 8.5	32.6± 4.5 33.1± 4.3	↓BMI *p* = 0.005; −1.5 ↓body mass *p* = 0.000;−4.09 ↓fat mass *p* = 0.000; −3.1	↓FBG *p* = 0.000; −0.28 ↓insulin *p* = 0.008; −1.84 ↓leptin *p* = 0.000; −3.63	↓SBP *p* = 0.000; −4.63 ↓DBP *p* = 0.002; −2.64
Razavi Zade M et al., (2016) [91]	DASH diet vs. Control group	Interventional study	n = 30 n = 30	8 week	39.7 ± 7.3 42.8 ± 10.6	28.5 ± 3.2 28.3 ± 3.3	↓BMI *p* = 0.01; −1.3 ↓body mass *p* = 0.006; −3.8	↓insulin *p* = 0.01; −3.3 ↓HOMA-IR *p* = 0.01; −0.8 ↑QUICKI *p* = 0.004; +0.02 ↓ALT *p* = 0.02; −8.4 ↓ALP *p* = 0.001; −26.3	↓TG *p* = 0.006; −31.3 ↓hs-CR *p* = 0.03; −1224.7
Mousavi SM et al., (2023) [106]	Moderately restricted carbohydrate diet vs. Control group	Interventional study	n = 35 n = 35	12 month	40.5 ± 6.4 40.1 ± 8.2	32.3 ± 3.8 32.4 ± 3.5	↓BMI *p* = 0.01; −1.88 ↓body mass *p* = 0.01; −4.82 ↓WC *p* = 0.01; −5.34 ↓HC *p* = 0.01; −2.58	-	↑HDL-C *p* = 0.01; +1.89 ↓TG *p* = 0.01; −26.8
Foroozanfard F et al., (2017) [97]	DASH diet vs. Control group	Interventional study	n = 30 n = 30	12 week	27.1 ± 4.7 25.6 ± 3.7	32.3 ± 4.6 32.2 ± 3.9	↓BMI *p* = 0.02; −1.6	↓insulin *p* = 0.02; −25.2 ↓HOMA-IR *p* = 0.02; −0.9 ↑QUICKI *p* = 0.02; 0.01	-
Nilghaz M et al., (2025) [92]	DASH diet vs. Control group	Interventional study	n = 21 n = 21	12 week	45.4 ± 11.5 46.1 ± 11.7	30.7 ± 5.6 31.9 ± 3.7	↓BMI *p* = 0.03; −2.92 ↓AC *p* = 0.005; −6.39	↓ALT *p* = 0.039; −15.2 ↓AST *p* = 0.047; −7.52	↓TG *p* = 0.049; −32.52
Monserrat-Mesquida M et al., (2022) [79]	Mediterranean Diet vs. Control group	Interventional study	n = 49 n = 48	24 month	64.5 ± 0.5 64.9 ± 0.4	33.2 ± 0.33 32.7 ± 0.3	↓WHR *p* = 0.033; −0.22	-	↓SBP *p* = 0.039; −5.1 ↓DBP *p* < 0.001; −6.6
Ebbeling CB et al., (2022) [108]	Low-carbohydrate diet vs. moderate-carbohydrate diet vs. high-carbohydrate diet	Interventional study	n = 53 n = 48 n = 46	20 week	35.7 for the whole group	32.2 ± 4.8 for the whole group	-	↓LPIR *p* = 0.009; −5.3 ↑adiponectin;	↑HDL-C *p* = 0.04; +0.09 ↓TG *p* = 0.006; −9.2 ↓TRL-P *p* = 0.001; +0.15 ↓Lp(a) *p* = 0.0005; −14.7
Sharifi M et al., (2024) [112]	Ketogenic diet vs. portfolio Moderate-carbohydrate diet	Interventional study	n = 19 n = 21	8 week	30.3 ± 5.4 30.1 ± 7.3	29.2 ± 3.4 29.5 ± 4.3	↓BMI *p* < 0.05; −2.9 ↓body mass *p* < 0.05; −5.64 ↓fat mass *p* < 0.05; −5.18 ↓lean mass *p* < 0.05; −3.19 ↓WC *p* < 0.05; −5.44 ↓HC *p* < 0.05; −7.31	↓FBG *p* < 0.05;−8.84 ↓insulin *p* < 0.05; −13.44 ↓HOMA-IR *p* < 0.05; −3.53	↓TC *p* = 0.03; −38.15 ↓LDL-C *p* < 0.05; −21.52 ↓TG *p* < 0.05; −61.42

WHR—waist-to-hip ratio; SBP—systolic blood pressure; DBP—diastolic blood pressure; BMI—body mass index; WC—waist circumference; FBG—fasting blood glucose; TCh—total cholesterol; LDL-C—low-density lipoprotein cholesterol; TG—triglycerides; HOMA-IR—homeostatic model assessment of insulin resistance; HDL—high-density lipoprotein cholesterol; hs-CRP—high-sensitivity C-reactive protein; IL-6—interleukin-6; HC—hip circumference; AST—asparagine transaminase; ALT—alanine transaminase; GGT—gamma-glutamyl transpeptidase; AC—abdominal circumference; QUICKI quantitative insulin sensitivity check index; ALP—alkalin phosphatase; VAT—visceral adipose tissue; ICO—index of central obesity; HbA1c—glycated haemoglobin concentration; LPIR—lipoprotein insulin resistance; TRL-P—triglyceride-rich lipoprotein particle; Lp(a)—lipoprotein(a); HOMA-B—homeostatic model assessment of Beta-cell function; PREDIM—predicted insulin sensitivity index; FBI—fasting blood insulin; ↑—increased, ↓—decreased.

**Table 2:** Effects of the analyzed observational studies in individuals with overweight and obesity.

Author (Year)	Type of Intervention/Diet	Type of Study	Sample Size (*n*)	Duration	Mean Age (Years)	Baseline BMI (kg/m^2^)	Anthropometric Outcomes	Glycemic & Metabolic Outcomes	Blood Pressure/Lipid Profile/Inflammation
Cicero AF et al., (2015) [105]	Very low-carbohydrate ketogenic diet in men vs. Very low-carbohydrate ketogenic diet in women	Observational study	*n* = 80 *n* = 297	12 month	48.3 ± 10.9 45.6 ± 9.9	32.1 ± 2.8 31.2 ± 3.1	↓BMI *p* < 0.001; −5.0 ↓fat mass *p* < 0.001; −8.1 ↓body mass *p* < 0.001; −14.0 ↓ICO *p* < 0.001; −0.8 ↓WC *p* < 0.001; −13.0	↓FBG *p* < 0.001; −8.7 ↓HbA1c *p* < 0.001; −0.3 ↓AST *p* < 0.001; −2.2 ↓ALT *p* < 0.001; −3.1 ↓GGT *p* < 0.001; −4.1	↓SBP *p* < 0.001; −10.5 ↓DBP *p* < 0.001; −2.2 ↓LDL-C *p* < 0.001; −19.5 ↑HDL-C *p* < 0.001; +3.5 ↓TG *p* < 0.001; −23.4
Farhadnejad H et al., (2019) [93]	DASH diet with tertile 1 vs. DASH diet with tertile 2 vs. DASH diet with tertile 3	Observational study	*n* = 1220 *n* = 1070 *n* = 928	*No data*	37.3 ± 9.0 39.6 ± 8.8 41.2 ± 8.9	29.5 ± 3.7 29.5 ± 3.9 29.7 ± 3.7	-	-	↑HDL-C *p* < 0.001; +0.9/+1.3

SBP—systolic blood pressure; DBP—diastolic blood pressure; BMI—body mass index; WC—waist circumference; FBG—fasting blood glucose; TCh—total cholesterol; LDL-C—low-density lipoprotein cholesterol; TG—triglycerides; HDL-C—high-density lipoprotein cholesterol; hs-CRP—high-sensitivity C-reactive protein; AST—asparagine transaminase; ALT—alanine transaminase; GGT—gamma-glutamyl transpeptidase; ICO—index of central obesity; HbA1c—glycated haemoglobin concentration; FBG—fasting blood glucose. ↑—increased, ↓—decreased.

**Table 3:** Mechanism of action and effects of bioactive components.

Bioactive Compound	Insulin Signaling Improvement	Lipid Profile Improvement	Anti-Inflammatory Effect	Antioxidant Effect	Gut Microbiome Modulation
Polyphenols	↑SCFAs production ↑GLP-1 secretion ↑insulin sensitivity ↓insulin resistance ↑satiety ↑fatty acid oxidation [122,123]	↓hepatocellular AMPK ↓adipogenesis ↓lipogenesis [124]	↑SCFAs production ↑anti-inflammatory mediators ↓pro-inflammatory cytokines [128]	↑SCFAs production ↑anti-inflammatory mediators ↓pro-inflammatory cytokines [129]	↑SCFAs production ↓pathogenic microbes count ↑beneficial bacteria count ↑gut microbiota barrier function [130,131]
Omega-3 fatty acids	↑SCFAs production ↑GLP-1 secretion ↑insulin sensitivity ↓insulin resistance ↑satiety ↑mitochondrial β-oxidation of fatty acids [133,135]	↓lipogenesis de novo ↓hepatic secretion of lipoproteins ↑apoB degradation [136,137]	↑SCFAs production ↑modifications to cell membrane lipid composition ↑anti-inflammatory mediators ↓pro-inflammatory cytokines ↓leukocyte activation and recruitment [138]	↓ROS generation ↓oxidant enzymes ↑scavenging superoxide [139,140]	↑SCFAs production ↓pathogenic microbes count ↑beneficial bacteria count ↑gut microbiota barrier function [141,142,143]
Dietary fiber/prebiotics	↑SCFAs production ↑GLP-1 secretion ↑insulin sensitivity ↓insulin resistance ↑satiety ↓gastric emptying [146,147,148]	↑SCFAs production ↓nutrient absorption [146,147,148]	↑SCFAs production ↓pro-inflammatory cytokines [146,147,148]	↓oxidant enzymes ↑antioxidant molecules [149,150,183]	↑SCFAs production ↓pathogenic microbes count ↑beneficial bacteria count ↑gut microbiota barrier function [151,152,182]
Probiotics/postbiotics	↑SCFAs production ↑GLP-1 secretion ↑insulin sensitivity ↓insulin resistance ↑satiety [157,158]	↑SCFAs production ↓pro-inflammatory cytokines [158]	↓pro-inflammatory cytokines [159]	↑antioxidant molecules ↑scavenging superoxide [159,160]	↑SCFAs production ↓pathogenic microbes count ↑beneficial bacteria count ↑gut microbiota barrier function [161,162]
Vitamin D	↑insulin sensitivity ↓insulin resistance ↑fatty acid metabolism [167,168]	↑fatty acid metabolism ↑adipose tissue metabolism [168]	↑anti-inflammatory cytokines ↓pro-inflammatory cytokines [169]	↓ROS generation ↓lipid peroxidation ↑antioxidant molecules ↑mitochondrial protection [170,171]	↑SCFAs production ↓pathogenic microbes count ↑beneficial bacteria count ↑gut microbiota barrier function [172,173]
Magnesium	↑SCFAs production ↑GLP-1 secretion ↑insulin sensitivity ↓insulin resistance ↑satiety [176,177]	↓lipogenesis [176]	↑SCFAs production ↓pro-inflammatory cytokines [176,177]	↓ROS generation ↑mitochondrial protection [176,177]	↑SCFAs production ↑gut microbiota barrier function [176,177]

SCFAs—short-chain fatty acids; GLP-1—glucagon-like peptide-1; AMPK—AMP-activated protein kinase; ROS—reactive oxygen species; apoB—apolipoprotein B. ↑—increased, ↓—decreased.

## Data Availability

No new data were created or analyzed in this study. Data sharing is not applicable to this article.

## Abbreviations

The following abbreviations are used in this manuscript:

AC	abdominal circumference
ALP	alkaline phosphatase
ALT	alanine transaminase
AMPK	AMP-activated protein kinase
apoA-I	apolipoprotein A-I
apoB	apolipoprotein B
BCAAs	branched-chain amino acids
BMI	body mass index
CE	capillary electrophoresis
DASH	Dietary Approaches to Stop Hypertension
DBP	diastolic blood pressure
DEXA	dual-energy X-ray absorptiometry
DHA	docosahexaenoic acid
DHEA	dehydroepiandrosterone
DSHEA	Dietary Supplement Health and Education Act
EFSA	European Food Safety Authority
EPA	eicosapentaenoic acid
FBG	fasting blood glucose
FBI	fasting blood insulin
FDA	Food and Drug Administration
FSH	follicle-stimulating hormone
FT-IR	fourier transform infrared spectroscopy
GC-MS	gas chromatography-MS
GLP-1	glucagon-like peptide-1
GLUT4	glucose transporter-4
HbA1c	glycated hemoglobin
HC	hip circumference
HDL-C	high-density lipoprotein cholesterol
HOMA-IR	homeostasis model assessment of insulin resistance
hs-CRP	high-sensitivity C-reactive protein
IL	interleukin
IR	insulin resistance
LC-MS	liquid chromatography-MS
LDL-C	low-density lipoprotein cholesterol
LH	luteinizing hormone
Lp(a)	lipoprotein(a)
LPIR	lipoprotein insulin resistance
MAFLD	metabolically associated fatty liver disease
MedDiet	Mediterranean Diet
MetS	metabolic syndrome
MHO	metabolically healthy obesity
MO	morbid obesity
MRI	magnetic resonance imaging
MS	mass spectrometry
MUO	metabolically unhealthy obesity
NF-κB	nuclear factor kappa B
NMR	nuclear magnetic resonance
PCOS	polycystic ovary syndrome
PUFAs	polyunsaturated fatty acids
RCT	randomized controlled trial
ROS	reactive oxygen species
SBP	systolic blood pressure
SCFAs	short-chain fatty acids
sdLDL	small, dense LDL
T2D	type 2 diabetes
TAC	total antioxidant capacity
TC	thigh circumference
TCh	total cholesterol
TGs	triglycerides
TNF-α	tumor necrosis factor-alpha
TRL-P	triglyceride-rich lipoprotein
VLDL	very-low-density lipoproteins
WC	waist circumference
WHO	World Health Organisation
WHR	waist-to-hip ratio
